# Projection of participant recruitment to primary care research

**DOI:** 10.1186/1745-6215-16-S2-P120

**Published:** 2015-11-16

**Authors:** David White, Daniel Hind

**Affiliations:** 1 University of Sheffield, Sheffield, UK

## Background

Recruitment to clinical trials remains challenging, particularly in primary care settings. Initial projections of participant recruitment should be as accurate as possible, in order to avoid the financial, clinical and ethical costs of trial extensions or failures. However, estimation of recruitment rates is challenging and often poorly executed. We used qualitative methods to explore the experiences and views of researchers on the planning of recruitment in this setting.

## Methods

Participants had registered accrual to a UK based primary care research study between April 2009 and March 2012. We interviewed ten chief investigators or study managers, using a semi-structured topic guide. Framework analysis was used.

## Results

1) A large number of factors affecting recruitment rates were identified. Use of targeted mail-outs was preferred where possible, eliminating some of the uncertainty involved where direct clinician referrals are required. 2) The importance of qualitative and pilot work were stressed, but we identified some ambivalence as to the benefits of formulating detailed recruitment projections, as well as uncertainty as to how best to schedule trial timelines to maximise efficiency. 3) Several potential sources of bias involved in the estimation of recruitment rates were identified, including technological, psychological and political factors.

## Conclusions

We found a large number of factors that impact on recruitment rates to primary care research, and highlighted the complexity of realistic estimation. The use of wider distributional information may improve accuracy when estimating accrual rates. Further research is needed to develop a formal approach to eliminate biases in recruitment projection.

